# Understanding the Contribution of HRM Bundles for Employee Outcomes Across the Life-Span

**DOI:** 10.3389/fpsyg.2019.02518

**Published:** 2019-11-15

**Authors:** Klaske N. Veth, Hubert P. L. M. Korzilius, Beatrice I. J. M. Van der Heijden, Ben J. M. Emans, Annet H. De Lange

**Affiliations:** ^1^Institute of Business Management, HRM, Hanze University of Applied Sciences, Groningen, Netherlands; ^2^Institute for Management Research, Radboud University, Nijmegen, Netherlands; ^3^Faculty of Management, Science & Technology, Open University of the Netherlands, Heerlen, Netherlands; ^4^Faculty of Economics & Business Administration, Ghent University, Ghent, Belgium; ^5^School of Management, Hubei University, Wuhan, China; ^6^School of Management, Kingston University, Kingston upon Thames, United Kingdom; ^7^Faculty of Economics & Business, University of Groningen, Groningen, Netherlands; ^8^Institute of HRM, HAN University of Applied Sciences, Nijmegen, Netherlands

**Keywords:** Human Resource Management (HRM), HRM bundles, employee outcomes, job demands, job resources

## Abstract

Using the Job Demands-Resources model literature and the life-span theory as scholarly frameworks, we examined the effects of job demands and job resources as mediators in the relationship between bundles of used HRM practices and employee outcomes. In addition, we tested for age differences in our research model. Findings confirmed the hypothesized original 2-factor structure representing maintenance and development HRM practices. Structural Equation Modeling analyses showed that the maintenance HRM bundle related directly and negatively to employee outcomes, without moderating effects of age. However, job resources appeared to mediate this relationship in a positive way as it also did for the development HRM bundle. Whereas this study showed the ‘*driving power’* of the actual use of HRM bundles through job resources, regardless of the employee’s age, this study also suggests a ‘*dark side’* of HRM. In particular, we found that development HRM bundles may also increase job demands, which, in turn, may result in lower levels of beneficial employee outcomes. These empirical outcomes demonstrate the strength of the *driving power* eliciting from job resources preceded by any HRM bundle. Moreover, this effect appears to apply to employees of all ages. Our moderated-mediation model appeared robust for several control variables. Overall, this study provides an extension of the well-known Job Demands-Resources model by including maintenance and development bundles of HRM practices used by employees that have a differential effect on job demands and job resources which in turn have an impact on employee outcomes.

## Introduction

Previously, ample Human Resource Management (HRM) research has paid attention to the effects of HRM on individual employee attitudes and behavior (e.g., Kuvaas, 2008; Van de Voorde et al., 2012; Pak et al., 2018). After all, it is through employees’ attitudes and behavior that organizational competitive advantage can be gained (Paauwe et al., 2013; De Vos et al., 2018). To better understand *how* HRM contributes to shape employees’ attitudes and behavior, additional research is needed to examine possible mediating linkages between HRM implementation and employee outcomes (see also Bowen and Ostroff, 2004). For that reason, and in line with previous suggestions by scholars in the field (Van de Voorde and Boxall, 2014; Albrecht et al., 2015), the Job Demands-Resources model (Demerouti et al., 2001b; Bakker et al., 2003) is used to investigate the influence of HRM on employee outcomes, through the hypothesized mediating role of work-related characteristics. Hence, this study extends the relationships between demands/resources and employee outcomes by adding the more distant-like HRM as a foundation of the more closely related demands and resources.

The aforementioned relationships occur in the context of an aging labor force. According to life-span theory, aging is generally associated with both gains and losses (Baltes and Baltes, 1990; Kanfer and Ackerman, 2004), which are assumed to affect the relationship between HRM, work-related characteristics, and employee outcomes. Specifically, nowadays, organizations are facing the combination of a constant and rather low number of young employees, due to continuous low birth rates, and the significant extension of life expectancy at birth within European and other developed countries (Eurostat, 2013; Hertel et al., 2013). This might require adaptations in HRM strategies, such as career development and retirement policies (Hedge et al., 2006) in order to find ways to enable workers into a prolonged working life (De Lange et al., 2015). Life-span theories (Kanfer and Ackerman, 2004; Baltes et al., 2006; Barnes-Farrell and Matthews, 2007; Maurer, 2007) have shed light on changes in workers’ needs, which have implications for the specific need for HRM throughout their career. However, evidence on how relationships between aging and employee outcomes take shape is not unambiguous. This illustrates the complexity of the broad concept that aging comprises (Zacher et al., 2018). This draws attention to the question of how managers may further develop and maintain an aging and active workforce (Hedge et al., 2006; Shultz and Wang, 2011; Hertel et al., 2013).

To date, to the best of our knowledge, no study has addressed the impact of mediators upon the relationship between HRM and employee outcomes, together with the effect of age (Korff et al., 2009). This is an important omission, since evidence suggests that employees’ work characteristics, such as job resources, have an impact on their attitudes and behavior (e.g., Bakker et al., 2003) across the life-span. Considering these limitations, the contribution of our paper is fourfold. Firstly, we aim to extend the Job Demands-Resources model literature (Demerouti et al., 2001b; Bakker et al., 2003) by investigating the relationships between several bundles of HRM practices that are actually used by employees, work-related aspects, and employee outcomes. To investigate these relationships, the JD-R model is used to discern *mediating mechanisms*, such as job demands (i.e., mental and emotional load) and job resources (i.e., learning opportunities and support from the supervisor), that are provoked by HRM practices, and that ultimately give rise to employee outcomes. Secondly, findings from life-span developmental psychology have identified various systematic age-related changes in human functioning (Baltes et al., 2006; Barnes-Farrell and Matthews, 2007; Maurer, 2007), work-related attitudes, and motivation (Finegold et al., 2002; Kanfer and Ackerman, 2004). Therefore, age has been included as a *moderating factor* in our research model. Thirdly, ample research has examined relationships between employees’ *perceptions* of HRM practices and employee outcomes (Gould-Williams, 2007; Macky and Boxall, 2007; Kuvaas, 2008; Gellatly et al., 2009; Gong et al., 2009; Paauwe, 2009). However, these perceptions can inevitably vary from its actual use (Gratton and Truss, 2003; Conway and Monks, 2008; Nishii et al., 2008; Snape and Redman, 2010). To date, there are hardly any empirical studies investigating the *actual* use of HRM (for an exception see Bal and De Lange, 2014). In our view, this is an omission since the *actual* use of HRM might also have an effect on employee outcomes. Fourthly, by examining HRM *bundles*, we go beyond several HRM studies that have taken into account isolated HRM practices (see also Huselid, 1995; Kooij et al., 2014; Veth et al., 2015). Following MacDuffie (1995), HRM bundles are specified as sets of interrelated and internally consistent HRM practices that are aimed to contribute to more productivity and quality than each HRM practice in itself. By combining these four contributions, our empirical work comprises a unique compound of perspectives.

**FIGURE 1:**
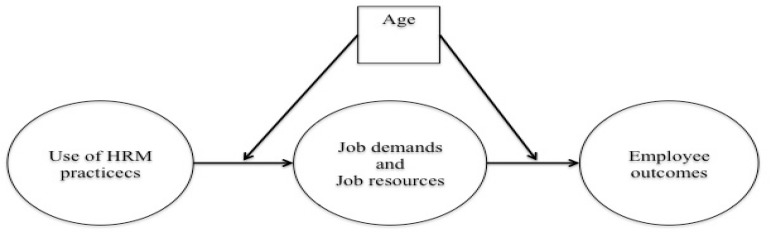
Research model.

Before elaborating on our theoretical framework, we explain our research setting. This study has been conducted in the Netherlands incorporating three working organizations from three different sectors: transport, healthcare, and research and education. Similar to many developed and developing countries, the Netherlands is confronted with unprecedented and rapid aging of populations and workforces alike (Ilmarinen, 2009; Chand and Tung, 2014). Furthermore, in order to warrant generalizability and to test the robustness of findings across occupational sectors, we explicitly included three distinctive sectors and strived for an equal distribution of both males and females, across age groups, from profit and not-for-profit organizations. Our research model, incorporating all hypothesized relationships, is presented in Figure 1.

## Extending the Job Demands-Resources Model Framework by Incorporating HRM

The research model for this study comprises an extension of the Job Demands-Resources model (JD-R) (Demerouti et al., 2001b; Bakker et al., 2003). According to the JD-R model, regardless of the specific occupation that is dealt with, two broad categories of work characteristics, can be distinguished: job demands (in this study mental load, emotional load, pace and amount of work) and job resources (in this study independence, learning opportunities, variety of work, support from supervisor, and support from colleagues). Job demands are those physical, social, or organizational aspects of the job that require sustained physical and/or psychological effort, and are, therefore, associated with physiological and/or psychological costs (Bakker et al., 2003, p. 344). Job resources refer to those physical, psychological, social, or organizational aspects of the job that: (1) may reduce job demands and the associated physiological and psychological costs; (2) are functional in achieving work goals, and: (3) stimulate personal growth, learning, and development (Bakker et al., 2003, p. 344). According to the JD-R model, job resources have motivating potential (Bakker and Demerouti, 2007, 2014), and the impact of work environments, characterized by many job resources, on employee outcomes has been widely acknowledged (Schaufeli and Bakker, 2004; Salanova et al., 2005; Llorens et al., 2007; Mauno et al., 2007; Hakanen et al., 2008). Our choice of outcomes variables is argued below.

The World Health Organization (2016) considers the following three elements as crucial for healthy work in which workers and managers collaborate to use a continual process to improve people’s well-being at work; (1) work engagement, (2) employability, and (3) health. First, as *work engagement* is also a central variable in the motivation process of the JD-R model that is expected to affect employee performance (Bakker and Demerouti, 2008), this variable is included as one of the employee outcomes in the present study. Work engagement is defined as a positive, fulfilling work-related state of mind that is characterized by vigor, dedication, and absorption (Schaufeli et al., 2002). Second, *employability* is also referred to as a positive outcome (Thijssen et al., 2008), and has been defined as ‘the capacity of continuously fulfilling, acquiring or creating work through the optimal use of competences’ (Van Der Heijde and Van Der Heijden, 2006, p. 453). Employability (or career potential) enables employees to cope with fast changing job requirements (Van Der Heijde and Van Der Heijden, 2006; Van der Heijden et al., 2009). Third, considerable previous research has shown the association between work characteristics and employee *health* (i.e., Wilson et al., 2004). As work is an influential part of employees’ lives, it also affects the quality of an individual’s life and his or her perceived mental health (Harter et al., 2002). Therefore, health is included as a third employee outcome. As argued by Van der Klink et al. (2016), and recently reconfirmed by Pak et al. (2018), these three employee outcomes are considered as aids to optimal functioning, and fit the recent trend to concentrate on optimal functioning.

Much previous empirical evidence suggests that work characteristics (job demands and resources) enhance employee outcomes, such as work engagement (Demerouti et al., 2001a, b; Salanova et al., 2005; Mauno et al., 2007). In this study, the JD-R model is extended with an examination of the impact of HRM on work characteristics, and, subsequently, on employee outcomes. In this regard, HRM is considered as a more distant-like provision of practices by the organization, whereas work characteristics are more closely related to the employee’s work(place), and therefore easier to influence. Earlier empirical evidence already suggests that work characteristics have an impact on employee’s attitudes and behaviors (e.g., Bakker et al., 2003), and therefore, we argue that they can mediate the relationship between HRM and employee outcomes. Hence, the underlying idea is that HRM is assumed to influence job demands and resources, which, may, subsequently, have an impact on employee outcomes. How these relationships manifest themselves exactly, depends on the character of the bundle of HRM practices.

The current empirical study examines the impact of two categories of bundles of HRM practices. We used an existing list of HRM practices (Kooij et al., 2010) that comprises all kinds of practices (ranging from management practices, such as job development interviews, to fringe practices, such as a sabbatical). Based on the results of a pilot study amongst HRM professionals, we added seven (age-related) HRM practices to the operationalization by Kooij et al. (2010), such as child care. In line with existing literature (e.g., Armstrong-Stassen and Schlosser, 2010; Kooij et al., 2014), we have first bundled HRM practices into sets of interrelated and internally consistent HRM practices aimed at achieving the same organizational purpose (MacDuffie, 1995). First, this distinction has been made conceptually in the theoretical framework, and thereafter confirmed using statistical analyses. The first category includes so-called maintenance HRM practices, referring to those practices that are aimed at retaining workers at their current level of functioning, or at recovery after a loss (Kooij et al., 2010). It is classified as the maintenance HRM bundle because of the shared goals of the specific HRM practices it entails, all being focused on preserving the *status quo*. Examples are part-time work and attention for health. The second category is called the development HRM bundle and consists of practices striving to reach higher levels of employee functioning. Regardless of the content of the specific HRM practices, all practices in this bundle are focused on growth, learning new tasks, and extending the employee’s horizon. Examples are continuous development and task enrichment.

However, in this study the focus lies on the actual *use* of HRM practices by employees, rather than on employees’ perceptions on intended or available HRM practices. We argue that it is insufficient to only shed light on employees’ perceptions about HRM (Gratton and Truss, 2003; Conway and Monks, 2008; Snape and Redman, 2010), because these can vary from its intentions and actual use (Nishii et al., 2008). Though it might be interesting to take into account the intentions behind the HRM practices at a strategic level, as reported by HRM professionals (e.g., Khilji and Wang, 2006; Nishii et al., 2008), or, employees’ perceptions of those HRM practices, in the end it all evolves around the *actual* use of those HRM practices by the employees (Legge, 1995; Keenoy, 1999; Purcell, 1999; Truss, 2001; Khilji, 2002; Lee and Allen, 2002) as the latter is accountable for real changes in employee outcomes.

Building upon the JD-R model (Demerouti et al., 2001b), we expected these two categories of HRM bundles to have differential effects on the work characteristics (job demands and resources) and, through these, on employee outcomes. Earlier scholarly work applying the JD-R model has already shown that job resources, such as independence in one’s work, social support from one’s supervisor and near colleagues, task variety, and opportunities to learn, are positively associated with employee outcomes. In a similar vein, job demands are defined as aspects of work that require effort. Hence, job demands are associated with costs and in line with the JD-R model assumed to negatively affect employee outcomes (Schaufeli and Bakker, 2004; Demerouti and Bakker, 2011). The rationale of how these mechanisms are related to the distinguished categories of HRM bundles is established below.

First, the maintenance HRM bundle is intended to result in diminishing job demands which will, subsequently, positively impact employee outcomes. For instance, working part-time allows employees to continue their career as well as to pursue their lives beyond the workplace (Collins, 2003), which will reduce mental load and, in the end, increase employee outcomes. Such an effect is reinforced by other practices such as flexible work conditions and telecommuting, altogether being ingredients of the maintenance HRM bundle.

Second, the relationship between the actual use of maintenance HRM practices and employee outcomes, through job resources, is assumed to have a negative character. For instance, ‘part-time work’ also implies a decreased amount of opportunities to learn (job resources), given the reduced presence at the workplace for the specific employee, and, in turn, impacts employee outcomes negatively.

Third, the so-called development HRM bundle contains a group of interconnected HRM practices that have the capacity to make work more resourceful (Van De Voorde et al., 2016). For instance, the development HRM bundle is focused on growth and expanding horizons, and may therefore be expected to increase job resources, such as support from one’s supervisor. Hence, we expect that practices being part of the developmental HRM bundle result in increased job resources, which, in turn, will positively impact employee outcomes.

Fourth, this development HRM bundle may also make the employee’s work more challenging and intense (Van De Voorde et al., 2016). For example, the use of training implies a time-consuming effort and, therefore, may lead to increased job demands, such as mental load, and is subsequently assumed to result in decreased employee outcomes. Hence, we believe that, unfortunately, HRM can also have ‘a dark side,’ which is in line with some earlier scholarly research (Jensen et al., 2013). Specifically, research related to enhancing employee skills, that is, increasing their motivation and facilitating empowerment (Wright and Boswell, 2002), showed results of employees feeling being exploited. For instance, attending training programs can be at the expense of employee’s time and energy and can therefore also lead to increased working pressure. These relationships, on the one hand, from maintenance HRM, through a decreased perception of job resources, to diminished employee outcomes, and, on the other hand, from development HRM, through an increased perception of job demands, to decreased employee outcomes, are the side effects of HRM. However, in general, HRM is acknowledged to be an important function that adds value to employee attitudes and behavior (Snape and Redman, 2010), by reducing job demands (enforced by maintenance HRM) and by improving job resources (enforced by development HRM). Following this line of reasoning, we hypothesized the following:

Hypothesis 1a: The positive relationship between the use of the maintenance HRM bundle and employee outcomes is (partially) mediated by job demands.
Hypothesis 1b: The positive relationship between the use of the maintenance HRM bundle and employee outcomes is (partially) mediated by job resources.
Hypothesis 1c: The positive relationship between the use of the development HRM bundle and employee outcomes is (partially) mediated by job demands.
Hypothesis 1d: The positive relationship between the use of the development HRM bundle and employee outcomes is (partially) mediated by job resources.

## Extending the JD-R Model Framework by Incorporating Life-Span Theory

Since employees have to work longer, due to an increased retirement age (Hedge and Borman, 2012), it is of utmost importance to examine the role of age in the relationship between HRM bundles and employee outcomes. Though there is voluminous literature that addresses the relevance of single or bundled HRM practices (e.g., Huselid, 1995; Becker and Gerhart, 1996; Delery and Doty, 1996; Guest, 1997, 2002; Paul and Anantharaman, 2003; Wright et al., 2005; Schuler and Jackson, 2007), up to now, only a few researchers have conducted empirical research on how age affects the relationship between HRM and important outcomes (e.g., Hedge et al., 2006; Staudinger et al., 2008). Building upon life-span theories, previously, scholars have identified some basic changes as people age (e.g., Baltes et al., 2006; Barnes-Farrell and Matthews, 2007; Maurer, 2007; Bal and De Lange, 2014). Therefore, changes in work-related attitudes and work motivation tied to life-span are to be expected (e.g., Kanfer and Ackerman, 2004).

According to life-span theorizing (Baltes et al., 2006), human development goes along differential trajectories that entail gains on some dimensions of human behavior and stability and/or losses on other dimensions. The so-called SOC model (see also Baltes and Baltes, 1990; Baltes and Carstensen, 1996) posits that successfully aging people *Select* subjectively their goals, *Optimize* their strategies for goals attainment, and *Compensate* for age-related losses. This process of adaptation is a dynamic, life-long development in which people and their environment mutually influence each other (Baltes, 1987). This age-dynamic proposition is supported by Ebner et al. (2006), who found, that as people age and have increasingly to deal with losses, for instance as regards their fluid intelligence, their goal focus shifts gradually from a predominant growth orientation to a goal focus on maintenance and prevention. Indeed, in their meta-analysis of 86 studies, Kooij et al. (2011) revealed that work-related motives change with age, specifically, from a stronger focus on extrinsic growth-related motives among younger workers to more intrinsic work-related motives for older workers. Therefore, in line with life-span theorizing, we argue that the impact of specific HRM practices or bundles may be dependent on the age of the involved employees (Ng and Feldman, 2008). Since the above-mentioned losses (for example in occupational competences) specifically occur in older age, SOC theory argues that the allocation of maintenance HRM or job demands used for maintenance and regulation of loss (i.e., compensation) will increase with age, whereas the provision of development HRM or job resources aimed at growth (i.e., optimization) will decrease with age (Baltes et al., 1999). These changing employee needs affect the actual use of HRM bundles, and therefore a moderating role of age is expected. More specifically, we hypothesized the following:

Hypothesis 2: The (partially) mediated relationship between the use of both the maintenance and the development HRM bundles respectively, on the one hand, and employee outcomes, on the other hand, through job demands and job resources, is moderated by age, such that the higher the age, the stronger the relationship in case the maintenance HRM bundle is the predictor (2a), and the weaker the relationship in case the development HRM bundle is the predictor (2b).

## Materials and Methods

### Respondents and Procedure

The data collection was done by means of an on-line questionnaire among 6,000 employees working in three Dutch working organizations from three different occupational sectors: transport, healthcare, and research and education. The questionnaires were distributed using the web-based tool Qualtrics Labs Inc. (2012), and were sent to all employees including the ones working as managers. The respondents were assured confidentiality, were informed about the added value of the research, and were offered some rewards in recognition of their participation. Initially, a total of 2,240 workers responded to the survey, representing a response rate of approximately 20%. Data from respondents who did not complete the whole questionnaire were list-wise excluded from further analyses. This resulted in a total sample of 1,121 respondents measuring age as a continuous metric variable in order to have more statistical power (i.e., establishing existing effects by means of the statistical tests) and to be able to comprehensibly study age as moderator in the various SEM analyses. A missing value analysis showed no significant differences between the data set of included and excluded respondents for gender, part-time versus full-time workers, age [χ^2^(1) = 0.13, *p* = 0.72; χ^2^(1) = 0.50, *p* = 0.48; χ^2^(1170) = 0.31, *p* = 0.76]. However, the category of respondents contained relatively more higher educated employees compared to the group of excluded ones [χ^2^(6) = 27.56, *p* < 0.001]. The mean age of the respondents was 46.9 years (*SD* = 10.2) and their mean job tenure was 8.54 (*SD* = 8.88). Please, see Table 1 for an overview of sample characteristics.

**TABLE 1:** Characteristics of the sample.

**Variable**	***n* (%)**
**Gender**	1152
Male	311 (27.0)
Female	841 (73.0)
**Marital status**	1167
Unmarried	118 (10.1)
Married/cohabiting/partnership	961 (82.3)
Divorced	79 (6.8)
Widowed	9 (0.8)
**Children**	1162
Yes	903 (77.7)
No	259 (22.3)
**Highest completed education**	1171
Elementary school	5 (0.4)
Lower vocational education	47 (4.0)
Secondary school	149 (12.7)
Secondary vocational education	370 (31.6)
Higher vocational education	259 (22.1)
Academic education	291 (24.9)
Other	50 (4.3)
**Contract**	1158
Part-time	842 (72.7)
Full-time	316 (27.3)
**Management**	1152
Line/staff/project	243 (21.1)
Non-management	909 (78.9)

### Measures

#### Employee Outcomes

The Utrecht Work Engagement Scale (UWES) developed by Schaufeli et al. (2002) was used to measure work engagement. This 9-item questionnaire measuring three components, vigor (3 items: e.g., ‘When I get up in the morning, I feel like going to work’), dedication (3 items: e.g., ‘I am enthusiastic about my job’), and absorption (3 items: e.g., ‘When I am working, I forget everything else around me’), was rated on a seven-point Likert-scale ranging from 1 (‘never’) to 7 (‘always’). The UWES subscales have shown acceptable levels of internal consistency with McDonald’s omega^1^ of 0.79, 0.81, and 0.81, respectively. We computed an overall work engagement score, as was recommended by Schaufeli et al. (2006; McDonald’s omega = 0.94).

The Employability instrument developed by Van Der Heijde and Van Der Heijden (2006; see also Van der Heijden et al., 2009) was used to measure employability. The 47-item list measuring five components, occupational expertise (15 items: e.g., ‘I consider myself competent to engage in in-depth, specialist discussions in my job domain’), anticipation and optimization (8 items: e.g., ‘How much time do you spend improving the knowledge and skills that will be of benefit to your work?’), personal flexibility (8 items: e.g., ‘How easily would you say you can adapt to changes in your workplace?’), corporate sense (7 items: e.g., ‘I am involved in achieving my organization’s/department’s mission’), and balance (9 items: e.g., ‘I suffer from work-related stress’), was rated on a six-point Likert-scale ranging from 1 (‘not at all’/‘very badly’/‘very little’/‘never’) to 6 (‘extremely’/‘a considerable degree’/‘very well’/‘a very great deal’/‘very often’). The employability instrument has shown acceptable levels of internal consistency with McDonald’s omegas of 0.91, 0.83, 0.77, 0.86, and 0.83, respectively. McDonald’s omega of the overall employability scale was 0.93.

The SF-36 Health-scale developed by Ware and Sherbourne (1992) was used to measure *general health perception*. The 5-item questionnaire (e.g., ‘I am as healthy as anybody I know’) was rated on a five-point Likert-scale ranging from 1 (‘definitely false’) to 5 ‘definitely true’). The SF-36 Health–scale has shown an acceptable level of internal consistency with a McDonald’s omega of 0.74.

### Job Demands

The Mental load and Emotional load, and the Pace and amount of work scales developed by Van Veldhoven et al. (2002) were used to measure job demands. The first two 7-item instruments (mental load e.g., ‘Does your work demand a lot of concentration?’ and emotional load e.g., ‘Does your work demand a lot from you emotionally?’) were rated on a four-point Likert-scale ranging from 1 (‘never’) to 4 (‘always’). The latter 11-item instrument pace and amount of work (e.g., ‘Do you have to work fast?’) was rated on a four-point Likert-scale ranging from 1 (‘never’) to 4 (‘always’). All three scales have shown acceptable levels of internal consistency with McDonald’s omegas of 0.87, 0.77, and 0.83, respectively.

### Job Resources

The scales Independence in your work, Opportunities to learn, and Variety in your work developed by Van Veldhoven et al. (2002) were used to measure job resources. The 11-item instrument of Independence in your work (e.g., ‘Do you have freedom in carrying out your work activities?’), the 4-item instrument of Opportunities to learn (e.g., ‘Do you learn new things in your work?’), and the 6-item instrument of Variety in your work (e.g., ‘Is your work varied?’) were rated on a four-point Likert-scale ranging from 1 (‘never’) to 4 (‘always’), and from 1 (‘not at all’) to (‘very much’). All three scales have shown acceptable levels of internal consistency with McDonald’s omega’s of 0.91, 0.83, and 0.75, respectively. In addition, the scales of Social support from the supervisor and Social support from colleagues developed by Van der Heijden (2002, 2003) were used to measure job resources. Both 4-item instruments (e.g., ‘Is your immediate supervisor able to appreciate the value of your work and its results?’ and ‘Do your immediate colleagues give you supportive advice?’) were rated on a six-point Likert scale ranging from 1 (‘never’/’absolutely not’) to 6 (‘very often’/’to strong degree’). Both scales have shown good levels of internal consistency with McDonald’s omega’s of 0.83 and 0.80.

### HRM Bundles

To identify commonly examined HRM practices that are in use by employees in the involved organizations, we first identified 21 relevant age-related HRM practices, in line with Kooij et al. (2010). In addition, seven age-related HRM practices were included: flexible work, telecommuting, attention for health, sport facilities, child care, paid parental leave, and paid care leave. Based on a pilot-study amongst six HRM professionals who agreed that all items were unambiguous and understandable as regards their wording with the Dutch version of Kooij et al. (2010) for our target group of respondents, the final list of HRM practices was established (see Appendix). For measuring the respondent’s use of HRM practices, preliminary questions were asked first about the availability of those practices. Subsequently, for practices that were said to be available, the respondents were asked whether they actually made use of them.

### Control Variables

We used job duration (in years), gender (male/female), sector (transport, healthcare, and research and education), and management position (yes or no) as control variables in our analyses.

### Statistical Analysis

The statistical analyses were performed with IBM SPSS Statistics (Version 25; Field, 2018) and IBM SPSS AMOS (Version 25; Arbuckle, 2006). First, we performed a confirmatory factor analysis (CFA) using maximum likelihood estimation on the latent variables (Byrne, 2010). Considering the large number of items (143) and thus the number of parameters that need to be estimated in relation to the number of respondents (Myers et al., 2011), we started with CFAs for the separate constructs. Based on Kooij et al. (2010), we assumed a 2-factorial structure of the 28 HRM practices. The other latent variables were considered to be 1-factorial.

Second, we developed a factor measurement model with all concepts combined that formed the basis of the structural equation model (SEM) used for testing the hypotheses. In that process, we tested several models to decide which was the best final models taking into account the covariance between the latent factors (Wang and Wang, 2012). We started we a basic factor model and iteratively increased its complexity. As with increasing model complexity the fit deteriorated, we stopped after five models. These outcomes are likely to happen as the ratio of sample size to the number of variables in all these models will gradually lower, and will result in suboptimal fit indices. Still, we then checked whether the HRM bundles’ data would be better estimated as a bi-factor model, where the HRM bundle is considered a general factor and the other factors are separately specified (Black et al., 2015), or whether these concepts should be seen as a factor model with formative rather than reflective indicators (Diamantopoulos et al., 2008).

In the CFA’s we used modification indices to determine which error terms could be related. Items with non-significant factor loadings were deleted. We assessed the absolute, approximate, and incremental fit indices (Marsh et al., 2004; Kline, 2005; Schreiber et al., 2006; Byrne, 2010). In particular, we used the absolute fit indices Normed Chi-square statistic (χ^2^/*df*) and the Standardized root mean residual (SRMR), the approximate fit index Root mean square error of approximation (RMSEA), and the comparative fit indices Comparative fit index (CFI) and Tucker-Lewis Index (TLI). The following cut-off values of these fit indices were used to assess the model fit: χ^2^/*df* < 2 or 3, SRMR and RMSEA < 0.08, CFI and TLI > 0.90 or 0.95. In addition, the Akaike information criterion (AIC) was used to evaluate different models in which smaller values indicate a better fit. In addition, we assessed convergent and discriminant validity based on the following criteria (Fornell and Larcker, 1981; Hair et al., 2010): Construct reliabilities ≥ 0.70, Average Variance Extracted (AVE) > 0.50, square root of AVE has to be greater than any of the construct inter-correlations. We also examined measurement quality by checking factor loadings and cross-loadings (Hair et al., 2010; McNeish et al., 2018).

Third, we calculated factor scores (see below) and carried out descriptive analyses and Pearson correlational analyses and partial correlations controlling for age.

Fourth, to test the hypothesized mediation effects for job demands and job resources in the relationship between maintenance and development HRM bundles, on the one hand, and employee outcomes, on the other hand (H1a-d); and the moderation effect of age in these relationships (H2a-b), we computed a moderated meditation SEM model. Given the issues with the sample size to variables ratio, we reduced the complexity of the hypothesized SEM model (i.e., number of free estimated parameters) by using manifest variables (see Jöreskog and Sörbom, 1993). To study the moderation effects of age, after centering, interaction terms of latent variables with age were computed. Following Schneider et al. (2005) and James et al. (2006), we started with a complete mediation model connecting all mediating variables to independent and dependent variables, and iteratively deleted non-significant paths. The indirect effects of HRM bundles via job demands and job resources on employee outcomes were assessed with a bias-corrected bootstrapping method of 1000 bootstrap samples that generated 95% confidence intervals (CI) and standard errors (Byrne, 2010). In case the 95% CI range did not include zero, an indirect effect was present (Wang and Wang, 2012). Next, for all significant effects that appeared after mediation, we included age as a moderating variable and iteratively deleted non-significant effects. We also reported effect sizes for proportions of explained variances for mediating and outcome variables, 0.02 small, 0.15 medium, and 0.25 large (Cohen, 1992).

Fifth, multigroup SEM analyses (Byrne, 2010) were employed to gauge the influence of the control variables effects of job duration (≤5 years, >5 years), gender (male, female), sector (healthcare, research, and education), and management position (no, yes). Finally, we tested a model in which the control variables were included as independent variables affecting job demands, job resources and employee outcomes. In the fourth and fifth stages, we employed the fit criteria described above. In all statistical analyses, alpha was set 0.05.

## Results

### Confirmatory Factor Analysis

**FIGURE 2:**
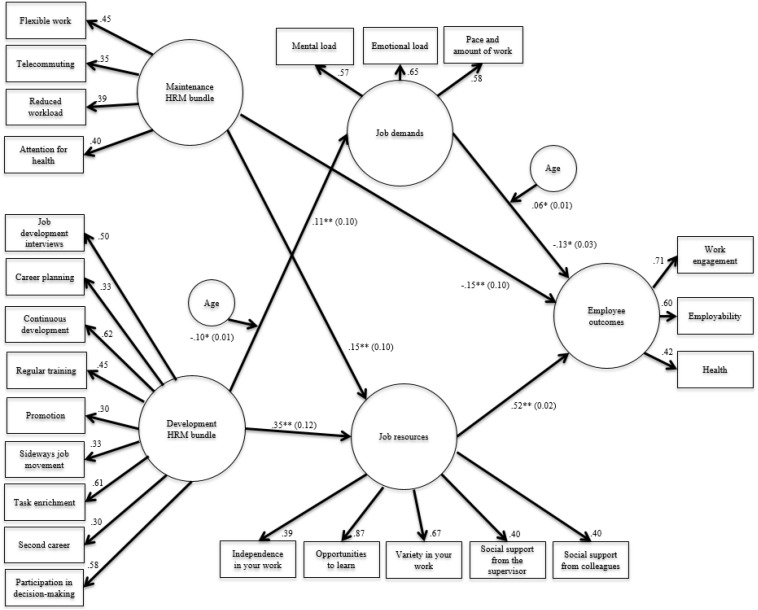
Mediation model of demands and resources in the relationships of HRM bundles and employee outcomes moderated by age. Values indicate standard loadings from the latent variables to the items (McNeish et al., 2018). In addition, we reported β coefficients and SE in brackets based on a SEM-model using manifest variables (see text).

Table 2 (first five rows) shows that the separate CFAs resulted in a good fit for the HRM bundles and for job resources. For job demands and employee outcomes, the models were just-identified (Hair et al., 2010, *p*. 699) and the fit could not be established. All standardized factor loadings were statistically significant (*p* < 0.001), ranged between 0.30 and 0.87, and 11 (out of 24) were 0.50 or higher (see also Figure 2), there were no cross-loadings. Convergent validity estimates were acceptable. Construct reliabilities were good for all latent variables, between 0.74 and 0.93. AVE was good for all latent variables but just below the threshold for job resources. Discriminant validity was good, that is, for all latent variables the criterion was met. In sum, the psychometric qualities of the latent variables were adequate.

**TABLE 2:** Confirmatory factor analyses of latent variables (*N* = 1121).

**CFA model**	**CR**	**AVE**	***χ2***	***df***	***χ2/df***	**AIC**	**SRMR**	**RMSEA**	**CFI**	**TLI**
Maintenance HRM bundle	0.82	0.53	189	58	3.27	255	0.04	0.04	0.94	0.92
Development HRM bundle	0.93	0.62								
Job demands	0.89	0.73	^a^							
Job resources	0.80	0.47	10.2	3	3.38	34.2	0.02	0.05	0.99	0.98
Employee outcomes	0.74	0.50	^a^							
Model 1			1452	65	22.3	1504	0.16	0.14	0.55	0.46
Model 2			2320	249	9.32	2422	0.12	0.09	0.60	0.56
**Model 3**			**1967**	**246**	**8.00**	**2075**	**0.11**	**0.08**	**0.67**	**0.63**
Model 4			3772	400	9.43	3903	0.14	0.09	0.65	0.62
Model 5			4161	399	10.4	4293	0.13	0.09	0.61	0.58
Model 6			1714	233	7.36	1848	^b^	0.08	0.71	0.66
Model 7			3721	263	14.15	3795	0.15	0.13	0.33	0.30

*Model 1, one factor: maintenance, development, job demands, job resources, employee outcomes. Model 2, one factor: HRM bundles maintenance and development, job demands, job resources, employee outcomes. Model 3, second order HRM bundles; one factor: job demands, job resources, and employee outcomes. Model 4, second order HRM bundles; one factor: job demands, job resources, health; second order: work engagement, and employability. Model 5, second order HRM bundles; one factor: job demands, job resources; third-order of second order factors work engagement and employability, and first order health. Model 6, a bi-factor model with a general HRM bundle (Black et al., 2015) and one factor: job demands, job resources, and employee outcomes. Model 7, a formative factor model of the HRM bundles maintenance and development (Diamantopoulos et al., 2008) and one factor: job demands, job resources, and employee outcomes. Measurement model is printed in bold. See Figure 2 for the operationalization of variables. CR, construct reliability; AVE, average variance extracted; AIC, Akaike information criterion; SRMR, Standardized Root Mean Residual; RMSEA, Root-mean-square error of approximation; CFI, comparative fit index; TLI, Tucker-Lewis index. ^*a*^Just-identified model, which “have a perfect fit by definition, meaning a fit assessment is not meaningful” (Hair et al., 2010, p. 690). ^*b*^Could not be calculated.*

The analyses confirmed maintenance and development as two distinctive factors. Of the 28 first-order items, 13 (i.e., HRM practices) loaded significantly on their intended dimension, the maintenance and development HRM bundles. Four items loaded significantly on the maintenance bundle: Flexible work, telecommuting, reduced workload, and attention for health. Nine items loaded significantly on the development HRM bundle: job development interview, career planning, continuous development, regular training, promotion, sideways movement, task enrichment, second career, and participation-in- decision-making.

### Factor Measurement Model

In a series of models we tested the combined factor measurement model (Table 2, model 1-y). As expected, given the complexity of the models, the fit indices were not very good. A model comparison showed that model 3 had the best fit (the combined factor model with second order factor HRM bundles containing first order factors maintenance and development, and one factor for: job demands, job resources, and employee outcomes). For the bi-factor model 6 in Table 2, SRMR could not be calculated, but it had relative good fit indices, which were comparable to those of model 3. The formative model 7 in Table 2 produced a worse fit and had much standardized factor loadings than the reflective factor solution. We therefore concluded that the distinguished HRM bundles are best described as two distinguished but related factors, maintenance and development, that represent the usage of two types of HRM practices. The other factors, job demands, job resources, and employee outcomes can be considered as one factor constructs. This factor measurement model formed the basis of the structural model used for testing hypotheses. Composite scores were computed for the number of used HRM practices per bundle, expressed as a ratio varying from 0 to 1. For the other latent variables, weighted factor scores were used.

### Descriptive Results

Table 3 presents the means, standard deviations, and correlations among all model variables. For clarity purposes, we only showed the results pertaining to the latent variables, and the moderator age. The outcomes indicated positive significant relations between the latent variables and the development HRM bundle, and job resources. However, the maintenance HRM bundle and job demands showed non-significant relationships with employee outcomes. Age was only positively correlated with job demands. The partial correlations, controlling for age, revealed that the inter-correlations of latent variables were hardly affected by age (as neither the significance nor the direction of the relationships were changed).

**TABLE 3:** Descriptives and (partial) correlations among latent variables and age.

**Variable**	***M***	***SD***	**1**	**2**	**3**	**4**	**5**
(1) Maintenance HRM bundle	0.26	0.27	–	0.44^∗∗^	0.04	0.30^∗∗^	0.01
(2) Development HRM bundle	0.31	0.23	0.44^∗∗^	–	0.11^∗∗^	0.41^∗∗^	0.14^∗∗^
(3) Job demands	0.00	0.80	0.05	0.11^∗∗^	–	0.17^∗∗^	–0.05
(4) Job resources	0.00	0.90	0.28^∗∗^	0.41^∗∗^	0.17^∗∗^	–	0.46^∗∗^
(5) Employee outcomes	0.00	0.80	0.01	0.14^∗∗^	–0.05	0.46^∗∗^	–
(6) Age	46.9	10.2	0.04	0.01	0.10^∗∗^	–0.01	–0.00

*Max *N* = 1121. HRM, Human Resource Management. ^∗^*p* < 0.05, ^∗∗^*p* < 0.01. Below diagonal Pearson’s *r* correlation coefficients are printed, above diagonal partial correlations controlling for age.*

### Hypotheses Testing

On the basis of CFAs, we specified flexible work, telecommuting, reduced workload, and attention for health as indicators of the maintenance HRM bundle. Job development interviews, career planning, continuous development, regular training, promotion, sideways movement, task enrichment, second career, and participation in decision-making were taken as indicators for the development HRM bundle. In SEM, the two HRM bundles were allowed to be correlated.

Figure 2 presents the results for the moderated mediation model. The fit of the model was adequate: χ^2^/*df* = 70.5/17 = 4.15, AIC = 108, SRMR = 0.04, RMSEA = 0.05, CFI = 0.95, and TLI = 0.91. Effect sizes for proportions of explained variances for job demands, job resources, and employee outcomes were up to medium/large (0.02, 0.19, and 0.26, respectively). The maintenance HRM bundle was not significantly related to job demands, but was significantly negatively directly related to employee outcomes (β = −0.14, *p* < 0.01). The maintenance HRM bundle was also indirectly positively related to employee outcomes through job resources [β = 0.08, *p* < 0.01, 95% CI (0.05–0.11)]. The development HRM bundle was positively related to both job demands (β = 0.11, *p* < 0.001) and job resources (β = 0.35, *p* < 0.001), but did not have a direct significant effect on employee outcomes. This established a complete mediation effect of the development HRM bundle on employee outcomes [indirect effect β = 0.17, *p* < 0.01, 95% CI (0.13–0.20)].

The moderated mediated model showed that the maintenance HRM bundle did not have a significant direct on job demands and therefore could not establish mediation which means rejection of *Hypothesis 1a*. Next, there was a direct negative effect as well as an indirect positive effect of the maintenance HRM bundle, via job resources, on employee outcomes. The latter implies that no support for *Hypothesis 1b*, which assumed a positive relationship, was found in our data. The effects of the development HRM bundle appeared to be completely and positively mediated by job demands and by job resources, herewith supporting *Hypothesis 1c* and *d*.

Regarding the moderating effects of age in our assumed mediation model outlined above, we found some interesting results as well. The relationship from the maintenance HRM bundle, through job resources, to employee outcomes was not moderated by age, and therefore there was no evidence for *Hypothesis 2a*. However, we did find moderating effects of age relating to job demands (see also Figure 2). More specifically, age appeared to significantly negatively moderate the relationship between the development HRM bundle and job demands (β = −0.10, *p* < 0.05), and positively between job demands and employee outcomes (β = 0.06, *p* < 0.05). As age did not affect the relationship of the development HRM bundle, via job resources, on employee outcomes, *Hypothesis 2b* was partially supported.

### Control Variables

Table 4 shows the results of the multigroup analyses of the control variables job duration, gender, sector, and management position.^2^

**TABLE 4:** Subgroup model testing + model testing possible effects of controls.

**Groups**	***χ2***	***df***	***χ2/df***	**AIC**	**SRMR**	**RMSEA**	**CFI**	**TLI**
Job duration (years: 1 ≤ 5; 2 > 5)	267	34	7.87	343	0.13	0.11	0.61	0.36
Gender (male; female)	237	34	6.97	313	0.12	0.07	0.82	0.70
Sector (health; research and education)	237	34	6.97	313	0.10	0.08	0.79	0.66
Management (0 = no; 1 = yes)	167	34	4.92	243	0.05	0.06	0.87	0.78
Controls model	228	35	6.52	290	0.08	0.10	0.69	0.51

By and large, the outcomes pointed to fairly robustness of the moderated mediation model (Figure 2). Regarding the association between the maintenance HRM bundle, directly and indirectly, via job resources, influencing employee outcomes, it appeared that the direct effect was especially relevant for non-managers. Pertaining to the indirect effect of the development HRM bundle, through job resources and job demands, on employee outcomes, this was particularly present among employees with a job duration ≤5 years, males, employees in the healthcare sector, and non-managers. The moderation effect of age was characteristic among female employees and among those working in the research and education sector. The final model in which the control variables were included as independent variables affecting job demands, job resources and employee outcomes, showed that although there were some significant paths of the control variables (e.g., positive direct effects of management on job demands and job resources), the model fit was worse in comparison with the model in Figure 2 indicating that the control variables did not have a serious impact on the mechanisms described above.

## Discussion

Firstly, this study addressed the exploration of the mechanisms through which the actual use of HRM bundles, via job demands and job resources, influence employee outcomes. Secondly, due to an aging workforce (e.g., Shultz and Wang, 2011), we studied the effects of age on these aforementioned mechanisms. Thirdly, we investigated the actual use of HRM. And lastly, we went beyond studying HRM practices separately, by investigating bundles of HRM practices. Our results showed two distinct processes, in terms of mediation, one starting off from the maintenance and one from the development HRM bundles. The maintenance HRM bundle related directly negatively to employee outcomes, and indirectly through job resources in a positive way. The use of development HRM appeared to be positively related to job demands and to job resources and, subsequently, the latter to entail higher employee outcomes. Hence, our results suggest the ‘*driving power*’ of both maintenance and development HRM through job resources. However, from our analyses, we may conclude that the use of development HRM increases job demands as well, which, in turn, results in lower employee outcomes. This might refer to the so-called ‘*dark side*’ of HRM (Jensen et al., 2013), being an important outcome of our study.

Theoretically, our results were fully in line with the JD-R model assuming that job demands are associated with costs, and subsequently, relate negatively with employee outcomes. In a similar vein, we found a direct, negative relationship between maintenance HRM and employee outcomes. In particular, employees using practices from this bundle, such as flexible work conditions or attention for their health, might be encouraged to do so because of lower levels of perceived well-being, just to give an example. The build-and-broaden theory (Fredrickson et al., 2000) might help us to explain this relationship. People who are not feeling engaged are more prone to make use of HRM for the mere objective of retaining their current job. In other words, they use these practices mainly in order to survive at the labor market.

The mediating role of job resources in the relationship between maintenance and development bundles of HRM, on the one hand, and employee outcomes, on the other hand, stresses the importance of these resources. Regardless of which HRM bundle was taken as an antecedent for these resources, the latter do play an important role. This is in line with the theory of Conservation of Resources (COR; Hobfoll, 2001; Hobfoll and Shirom, 2001). At its core, COR theory is a motivational theory that focuses on protecting resources, gaining resources, and preserving resources. People employ key resources, not only to respond to stress, but also to build a reservoir of sustaining resources for times of future need. Specifically, COR theory states that people must invest resources in order to protect against resource losses, to recover from losses, and to gain resources. For example, one might invest in resources, such as increasing employees’ learning opportunities, in order to counteract losses of employee engagement, due to the lack of challenges they experience in their work.

From a theoretical perspective, the development HRM bundle appears to function as a ‘bipod.’ In general, the process from the development HRM bundle through job demands to employee outcomes, and from the development HRM bundle through job resources to employee outcomes shows similarities with the health impairment and motivational processes in the JD-R model, respectively (Demerouti et al., 2001b; Bakker et al., 2003). In a similar vein, as the JD-R model states that an investment in the growth of job resources is a more productive approach than an investment in the reduction of job demands (Bakker and Demerouti, 2008; Bakker et al., 2010; Veth et al., 2015), we may state that serious investment in both HRM bundles - accompanied with an investment in job resources - is rewarding. We find evidence that particularly job resources, regardless of which HRM bundle precedes these, elicits positive employee outcomes.

Further, this study showed hardly any moderating effects of age on the relationships between HRM bundles of practices and employee outcomes. Nevertheless, interactions of age with the HRM development bundle, through job demands, and employee outcomes, showed significant differences in employees across the life-span. High job demands appear to harm all employees, regardless of their age, yet appear to be more harmful (in terms of a decrease in positive work outcomes) for aging workers. Moreover, the so-called ‘dark side’ effect of HRM that was found for the impact of the development HRM bundle on job demands was weaker for the older workers as well. Apparently, a high amount of job demands are a risk factor for all employees, and as employees age, the use of development HRM bundle may have an increasingly supportive function. Kooij and Van de Voorde (2015) argued that since older workers are better at regulating their emotions, they are better able to deal with increased workload, to mention an example, resulting in weaker negative associations between development HRM bundle and employee outcomes. Notwithstanding this moderating effect, one ought to be always cautious for a possible so-called ‘dark side’ effect.

As regards the third and fourth contribution of this study it is unique in its integration of the aforementioned moderating mediating relationships together with the validation of an up to now conceptually distinction of HRM bundles, and the actual use of these. So, we go beyond the perceived availability of HRM, and investigated the actual behavior, the actual use of HRM and its’ relationships. Moreover, with this study we validated the conceptual distinction of Kooij et al. (2010) and validated these. So, this study points to consistent HRM practices forming a bundle targeting at higher levels of productivity and quality (MacDuffie, 1995).

To conclude, our results show a rather ambiguous effect of age on the relationship between development HRM and job demands (e.g., Kanfer and Ackerman, 2004; Zacher et al., 2018). However, in models with maintenance HRM as predictor we do not find an effect of age. This is opposed to predictions derived from the life-span theories (e.g., Baltes et al., 2006; Barnes-Farrell and Matthews, 2007; Maurer, 2007), and the SOC model framework (Baltes and Baltes, 1990). These theories state that gradual changes from a focus on growth to a focus on security occur as employees age, because of age-related losses. Apparently, the use of HRM may have ‘driving power’ for all employees regardless of age, resulting in higher employee outcomes through job resources, although one should never ignore the possible ‘dark side’ effect of the development bundle of practices.

### Limitations

The present study has some limitations. First, all data were collected using questionnaires, that may have resulted in biases like response set and common method variance. Second, all data were collected at one point in time, that is, the study was cross-sectional. Further research is therefore needed to address the issue of causality and reciprocal effects. As Taris and Kompier (2003) and De Lange (2005) already suggested, further research using multi-wave designs can provide more information about the stability and change of variables over time (i.e., cross-lagged designs). For instance, it might occur that, in particularly for older people, after a period of adverse health, employees may want to use more maintenance bundle HRM practices than before such of a period. Third and last, this study has been conducted in the Netherlands in three different working organizations from three occupational sectors, that are overrepresented by either males or females. The interpretation of HRM practices may be prone to cultural and sectorial biases. Nevertheless, the outcomes of our analyses provide robustness of findings across these three sectors. However, more research is needed to safely conclude on the generalizability of our results.

### Practical Implications

From a practical stance, the role of HRM in retaining employees’ well-being appears crucial in the realm of a continuously demanding working environment (e.g., Gould-Williams, 2007; Kuvaas, 2008; Gellatly et al., 2009; Gong et al., 2009; Taylor et al., 2012; De Lange et al., 2015). Therefore, in order to retain suitable workers for the labor market, across their working life-span, it would be helpful for an organization to know which HRM bundles should be targeted to distinctive age groups.

This study shows that age only plays a significantly distinctive role in relation to the effect of job demands in the modeled relationships. The predominant association between the both HRM bundles and employee outcomes, through job resources, turned out to be strongly positive, regardless of age. This outcome implies that, for all age categories of workers, the use of particularly development HRM bundle is highly important and should be stimulated by (HRM) managers. However, the unexpected direct negative relationship between the maintenance HRM bundle and employee outcomes was counterbalanced in case job resources were included as a mediator in this relationship.

All in all, this study indicates that stimulating the role of job resources (the so-called ‘driving power’ in our model), whether they are preceded by the use of a maintenance or by a development HRM bundle, is rewarding for employees of all ages. In addition, after controlling for job duration, gender, sector, and having a management position (yes or no), we found that the process of ‘driving power’ applies more to employees with a job duration longer than 5 years, for men, for the education and research sector, and for managers. The ‘dark side’ of HRM seems to be perceived more by the counterparts: employees with job duration shorter than 5 years, women, healthcare sector, and employees without a managerial position. Our study points out that it is highly relevant to understand which and how HRM bundles influence particular employees, and employee outcomes. Hence, this study questions the contribution of age-related HRM policies. However, HRM policies focused on particular employee groups like being a non-manager, or female, turns out to be important. Managers should use the job development interviews to deepen the understanding of how the dark side can be enlightened. They should find out what exactly contributes to the dark side, and what can be done to facilitate employees. As such, our approach may establish a foundation for further theory development, and empirical research on the topic of employee well-being from the perspective of HRM.

## Data Availability Statement

The datasets generated for this study are available on request to the corresponding author.

## Author Contributions

KV: overall text and research. HK: methodology and statistical analyses. BV: overall text and research. BE: theoretical foundation. AL: overall text.

## Conflict of Interest

The authors declare that the research was conducted in the absence of any commercial or financial relationships that could be construed as a potential conflict of interest.

## Footnotes

1 For scale reliabilities, we reported the McDonald’s omega rather than Cronbach’s alpha because it is a more robust method as it takes into account the possible differential impact of the various items of a scale (McNeish et al., 2018). Omega was calculated with the statistical package JASP (V. 0.10.2), University of Amsterdam.

2 The results of the analyses of the control variables will be provided upon request.

## Appendix

### List of HRM practices

**Table:**

(1) Part-time work	(8) Part-time retirement	(15) Continuous development	(22) Second career
(2) Compressed workweek	(9) Long career break (sabbatical)	(16) Regular training	(23) Participation in decision-making
(3) Flexible work	(10) Variable remuneration	(17) Promotion	(24) Attention for health
(4) Telecommuting	(11) Flexible labor conditions	(18) Demotion	(25) Sport facilities
(5) Additional leave	(12) Ergonomic adjustments	(19) Sideways job movement	(26) Child care
(6) Exemption from overtime working	(13) Job development interviews	(20) Task enrichment	(27) Paid parental leave
(7) Early retirement	(14) Career planning	(21) Reduced workload	(28) Paid care leave
